# Intracellular Signalling by C-Peptide

**DOI:** 10.1155/2008/635158

**Published:** 2008-03-18

**Authors:** Claire E. Hills, Nigel J. Brunskill

**Affiliations:** ^1^Department of Infection, Immunity and Inflammation, University of Leicester, Leicester School of Medicine, P.O. Box 138, Leicester LE1 7RH, England, UK; ^2^Department of Nephrology, Leicester General Hospital, Gwendolen Road, Leicester LE5 4PW, England, UK

## Abstract

C-peptide, a cleavage product of the proinsulin molecule, has long been regarded as biologically inert, serving merely as a surrogate marker for insulin release. Recent findings demonstrate both a physiological and protective role of C-peptide when administered to individuals with type I diabetes. Data indicate that C-peptide appears to bind in nanomolar concentrations to a cell surface receptor which is most likely to be G-protein coupled. Binding of C-peptide initiates multiple cellular effects, evoking a rise in intracellular calcium, increased PI-3-kinase activity, stimulation of the Na^+^/K^+^ ATPase, increased eNOS transcription, and activation of the MAPK signalling pathway. These cell signalling effects have been studied in multiple cell types from multiple tissues. Overall these observations raise the possibility that C-peptide may serve as a potential therapeutic agent for the treatment or prevention of long-term complications associated with diabetes.

## 1. INTRODUCTION

Historically proinsulin C-peptide has been regarded as an inert byproduct of insulin synthesis and processing. In the last decade, however, an enlarging body of evidence has emerged from several laboratories reporting both in vitro and in vivo effects of C-peptide of great potential relevance to the pathophysiology and treatment of diabetes. Observations that C-peptide may exhibit characteristics of a peptide hormone beneficially affecting nerve, renal and microvascular functions in diabetic animals and in patients with diabetes [1–7], have stimulated interest in the molecular mechanisms by which cell signals elicited by C-peptide are transduced into altered cell and organ behaviour.

## 2. A C-PEPTIDE RECEPTOR

Clearly if C-peptide has peptide hormone-like actions, it is necessary to postulate the existence of a receptor. Indeed, specific and displaceable binding of ^125^I-labelled C-peptide was first demonstrated by Flatt et al. [8] who derived a curvilinear Scatchard plot of specific C-peptide binding to pancreatic islet B-cells.

Subsequently, using retro sequence and all-D-amino acid C-peptide enantiomers, Ido et al. [9] suggested that C-peptide-induced improvements in nerve function and vascular permeability blood flow in diabetes through increases in Na^+^,K^+^-ATPase activity did not result from C-peptide binding in a stereospecific manner to a receptor. Instead it was hypothesised that biological activity of C-peptide was dependent on poorly defined membrane interactions that took place due to structural features related to the C-peptide sequence, but independent of its direction or chirality. A midportion sequence of C-peptide, largely conserved, and comprising a high proportion of nonpolar amino acids flanking a C16 proline was implicated in this activity.

Other investigators have also examined C-peptide fragment bioactivity using Na^+^,K^+^-ATPase activity in rat renal tubular segments as a readout [10]. Two segments of C-peptide demonstrated functional importance. The rat C-peptide carboxy terminal pentapeptide, EVARQ, elicited 100% of the activity of intact C-peptide whereas the remaining portion of the molecule, lacking this 5 amino acid sequence, was totally inactive. In this rat system, the terminal pentapeptide of human C-peptide (EGSLQ) elicited 75% activity; and notably the glutamic acid at position 1 and the glutamine at position 5 are generally conserved in mammals. Similar well-defined C-terminal functional sequences are also found in gastrin and cholecystokinin. Overall, the behaviour of the C-termimal pentapeptide in these studies was typical of a peptide ligand interacting with a specific receptor. In contrast, several C-peptide midregion sequences were able to partially recapitulate the activity of the intact molecule but exhibited rather different properties. Activity relating to this region was not seen with the des-(27–31)-C-peptide, and several nonnatural D-amino acid containing sequences showed some activity. The activity of these segments decreased if they were greater than 9 amino acids in length. This behaviour is not reminiscent of peptide-receptor interactions, but similar to the nonspecific type interactions of C-peptide with plasma membranes postulated by Ido et al. [9].

How the balance of activity provided by these two regions of the C-peptide molecule is manifest in vivo is not well understood. Very recent work suggests that efficient activation of signalling pathways requires the presence of conserved glutamic acid residues at positions 3, 11, and 27 of C-peptide, and the presence of helix-promoting residues in the N-terminal segment [11]. Taken together with the data relating to its carboxy-terminus, the overall picture now emerging of the structure-activity relationship of the C-peptide molecule is one of a tripartite structure where the terminal sections are involved in functional interactions, with the midregion forming a joining segment [11].

Analysis of rhodamine-labelled human C-peptide binding to a variety of human-cell membranes has now been carried out using highly sensitive fluorescence correlation microscopy, allowing measurement of membrane interactions at the single-molecule level in sub-fL volumes [12]. High-affinity specific binding of C-peptide was observed with an association rate constant of *∼*3 nM; half-maximal occupation of binding sites was seen at 0.3 nM C-peptide; and full occupation at 0.9 nM C-peptide. The maximal number of binding sites, approximately 1000–1500 per cell, was found on human renal tubular cell membranes. Binding could be displaced by excess intact C-peptide and by the C-terminal pentapeptide, but not by a randomly assembled scrambled C-peptide, insulin, and insulin-like growth factor (IGF)-I or IGF-II. Importantly, binding of C-peptide could also be largely inhibited by pertussis toxin (PTX) pretreatment of cells [12]. Overall, these results strongly support the existence of a specific GTP-binding protein-coupled receptor (GPCR) for C-peptide, linked either to the G-protein *α*-subunit G*α*_*i*_ or G*α*_*o*_, which interacts with the C-terminal penatpeptide region of the C-peptide molecule.

Attempts to identify a C-peptide receptor by gene-cloning strategies or using proteomic approaches have, to date, been unsuccessful. Luzi et al. recently reported their inability to isolate a C-peptide receptor either by screening a human lung fibroblast *λ*phage cDNA expression library or by proteomic analysis of proteins co-immunoprecipitated from C-peptide treated human fibroblasts using anti-C-peptide antibodies [13]. This information was published in the context of a short-debate paper and therefore methodological description was by necessity brief. Certainly this data does not prove that a receptor does not exist, and it remains perfectly possible however that alternative methodologies may successfully identify a C-peptide receptor in the future. Effort in this area is well justified and urgently needed.

## 3. C-PEPTIDE AND THE NA^+^,K^+^-ATPASE

Na^+^,K^+^-ATPase is an ubiquitous membrane-associated protein complex that uses energy from the hydrolysis of ATP to drive the counter-transport of sodium and potassium across the plasma membrane. It is widely accepted that impaired Na^+^,K^+^-ATPase activity is present in a variety of cell types in diabetes and contributes to the pathogenesis of diabetic complications [14–17]. The first indications that C-peptide may regulate the activity of intracellular enzymes came from studies of the Na^+^,K^+^-ATPase.

The kidney tubule is a particularly rich source of Na^+^,K^+^-ATPase and Ohtomo et al. described activation of this enzyme by rat C-peptide in rat kidney tubules at low to high nanomolar concentrations [18]. This effect was abolished by PTX and, being inhibited by the Ca^2+^-calmodulin-dependent protein phosphatase 2B (calcineurin) inhibitor FK506, appeared dependent on intracellular Ca^2+^ concentration. Interestingly the stimulatory effect of C-peptide on Na^+^,K^+^-ATPase in these experiments was substantially augmented by neuropeptide Y (NPY).

Extending these findings, the same group of investigators studied the ability of C-peptide fragments to activate Na^+^,K^+^-ATPase in rat kidney proximal tubule segments [10]. Altogether 36 different peptides and amino acids corresponding to various regions of intact C-peptide were investigated. Carboxy-terminal tetra- and penta-peptides elicited full activity whilst midregion peptides elicited only partial activity at best. These results were in broad agreement with those of Ido et al. [9] (discussed above) and those of the C-peptide binding studies published the following year [12], and, overall, they supported the nascent concept of C-peptide binding to a specific receptor with subsequent activation of downstream effector enzymes.

Motivated by these observations of C-peptide-induced increases in Na^+^,K^+^-ATPase activity and associated improvements in nervous system and renal function, it was hypothesised that the low C-peptide levels and reduced Na^+^,K^+^-ATPase activity in erythrocyte membranes observed in type 1 diabetes may be related. Accordingly, it was found that in patients with type 1 diabetes and complete C-peptide deficiency, erythrocyte activity Na^+^,K^+^-ATPase was consistently lower than healthy controls [19]. In type 2 diabetes, erythrocyte Na^+^,K^+^-ATPase was significantly lower in insulin-treated patients than in those treated with oral hypoglycaemic agents. Indeed the fasting C-peptide level was the only variable studied that independently correlated with Na^+^,K^+^-ATPase activity [19]. Subsequently, it was shown that infusion of C-peptide into patients with type 1 diabetes resulted in an increase in plasma cGMP and erythrocyte membrane Na^+^,K^+^-ATPase activity. A C-peptide dose response was evident in these studies where maximal effects were observed with achieved plasma C-peptide levels of *∼*3.5 nM [20]. Similar improvements in rat nerve Na^+^,K^+^-ATPase activity and function have also been observed after exogenous administration of C-peptide [9].

The mechanism(s) by which C-peptide activates Na^+^,K^+^-ATPase has been examined. Using isolated rat kidney medullary thick ascending limb tubules (MTALs) C-peptide was found to activate Na^+^,K^+^-ATPase at physiological concentrations [21]. In addition, treatment of MTALs with C-peptide resulted in phosphorylation of the Na^+^,K^+^-ATPase *α*-subunit and translocation of the Ca^2+^-dependent protein kinase C (PKC)-*α* to the membrane, an index of its activation. Both of these latter effects were blocked by an inhibitor of PKC [21].

Overall, the studies of Na^+^,K^+^-ATPase activity have delineated a stimulatory signalling action of C-peptide at physiological concentrations dependent on intracellular Ca^2+^ and PKC, and sensitive to PTX suggesting the presence of a GPCR for C-peptide. Replacement of C-peptide in diabetic animals and patients with diabetes has a salutary effect on Na^+^,K^+^-ATPase activity in a variety of tissues affected by diabetic complications.

Prompted by this evidence linking C-peptide to the Na^+^,K^+^-ATPase, and mindful that vasopressin also stimulates Na^+^,K^+^-ATPase activity, Maestroni et al. [22] studied C-peptide effects on a recently described vasopressin receptor; the vasopressin-activated calcium mobilising receptor (VCAM-1). They found that in human skin fibroblasts and human mesangial cells, C-peptide increased expression of VCAM-1 at both RNA and protein levels [22]. The effect was maximal with 1 nM C-peptide and inhibited by PTX. Although no direct link between C-peptide-induced increases in VCAM-1 and enhanced Na^+^,K^+^-ATPase activity was studied, enhancement of vasopressin action via upregulation of VCAM-1 provides a further mechanism for C-peptide action relating to the Na^+^,K^+^-ATPase.

## 4. THE EFFECT OF C-PEPTIDE ON ENDOTHELIAL NITRIC OXIDE SYNTHASE (eNOS)

One consistent observation following in vivo administration of C-peptide in type 1 diabetes is augmentation of microvascular blood flow to tissues and organs including muscle, skin, and kidney [23]. The possibility that these effects could be mediated by C-peptide regulation of nitric oxide (NO) was first provided by the finding that increased glucose utilisation in streptozotocin diabetic rats stimulated by C-peptide was sensitive to inhibition of NOS by coadministration of N-monomethyl-l-arginine (L-NMMA) [24]. This finding was in agreement with earlier studies that described an NO-dependent pathway for glucose transport and metabolism by muscle tissue [25, 26]. Subsequently, it was demonstrated that that C-peptide mediated arteriolar dilatation was dependent on NO [27, 28].

Details of the mechanism underlying C-peptide's ability to increase NO production have been provided by Wallerath et al. [29]. Working with a bovine aortic endothelial cell model (BAEC), these investigators reported that at the physiological postprandial concentration of 6.6 nM, C-peptide stimulated NO release consequential upon a rise in intracellular Ca^2+^. These authors speculated that C-peptide signalling resulted in activation of the Ca^2+^-sensitive endothelial NOS (eNOS) following increased Ca^2+^ influx, thus explaining in large part the vasodilatory effects of C-peptide observed in vivo [29].

Other workers have demonstrated upregulation of eNOS expression by C-peptide. In BAEC, enhanced NO release stimulated by C-peptide was accompanied by upregulation of eNOS gene transcription [30]. This effect appeared to be dependent on the upstream phosphorylation and activation of extracellular signal-regulated mitogen activated protein kinase (ERK).

Activation of the NO system by C-peptide signalling may also have other consequences. In C-peptide, injected rats expression of eNOS is increased as is basal aortic NO production. Reduced cell surface expression of the adhesion molecules P-selectin and ICAM-1 on the microvascular endothelium was observed and as a result leukocyte/endothelial interactions were attenuated [31].

## 5. STIMULATION OF MITOGEN ACTIVATED PROTEIN KINASES (MAPKs) BY C-PEPTIDE

The MAPKs are evolutionary conserved enzymes that link cell-surface receptors or chemical and physical stresses to key regulatory targets within cells [32–34]. MAPKs phosphorylate multiple targets on serine and threonine and, as a result, control many critical cell functions such as growth, gene expression, and cell survival and adaptation. The MAPK family includes the ERKs 1 and 2, c-Jun N-terminal kinases (JNK1, JNK2, JNK3), p38s (p38*α*, p38*β*, p38*γ*, p38*δ*), and ERK5.

In recent years, several reports have described the ability of C-peptide to induce phosphorylation and activation of members of the MAPK family. The initial impetus for study in this area is developed from the earlier observations of synergy between C-peptide and NPY signalling in the activation of Na^+^,K^+^-ATPase [18]. NPY is well known to activate MAPK [35, 36] and this finding therefore prompted Kitamura and colleagues to study possibility of MAPK mediated C-peptide signalling in the mouse embryonic fibroblast cell line, Swiss 3T3 [37]. Using both immunoblotting of phosphorylated ERK1 and ERK2 with phosphospecific antisera and an in vitro kinase assay as readouts of ERK activation, these workers showed that incubation of 3T3 cells with C-peptide resulted in brisk activation of ERK evident at a concentration as low as 1 pM and maximal with 1 nM C-peptide [37]. This stimulatory effect was also seen with NPY, and activation of ERK by both NPY and C-peptide was abolished by PTX. Neither retrosequenced nor all D-amino acid human C-peptide stimulated ERK. Not all cell types tested by these workers responded to C-peptide with ERK activation. No effect was seen in 3T3-L1 cells, L_6_E_9_ muscle cells, HepG2 hepatoma cells, NG108.15 neuroblastoma cells, or C6 glioma cells [37].

Activation of ERK is involved in eNOS gene transcription, an event previously also shown to be increased by C-peptide (see above). In an attempt to link these phenomena, studies were performed in LEII mouse lung capillary endothelial cells to examine the ability of C-peptide to activate transcription factors [38]. In this cell type, C-peptide stimulated both p38 and ERK MAPK activities, but not c-Jun N-terminal kinase. By comparison, insulin was only able to activate ERK but not p38 MAPK. In addition, C-peptide activated the cAMP response element (CRE)-binding protein (CREB)/activating transcription factor-1 (ATF-1) in a p38 but not ERK-dependent manner, thereby causing binding of these transcription factors to CRE. Consequently, it was confirmed that enhanced eNOS transcription in BAECs following C-peptide treatment was indeed MAPK-dependent [39]. Therefore differences in C-peptide responses between various cell types clearly exist. In BAECs, ERK activation is required for eNOS transcription, whereas transcription factor activation in LEII cells follows C-peptide stimulation of p38.

Loss of renal tubular cells is a prominent feature of diabetic nephropathy. It is therefore of significance that tubular cell growth and survival may be modulated by C-peptide signalling. In the opossum kidney (OK) immortalised proximal tubular cell line C-peptide also potently activates ERK, maximally at a concentration of 300 pM and declining thereafter with a bell-shaped dose-response curve [40]. Furthermore, C-peptide also induced activation of Akt in OK cells. This event was sensitive to wortmannin and indicative of phosphatidylinositide-3-kinase (PI-3-kinase) activation. The dose-response curve for Akt activation revealed a maximal effect at a C-peptide concentration of 5 nM, remaining constant thereafter up to a C-peptide concentration of 100 nM, and thus being quite distinct from that for ERK activation. In addition, C-peptide caused an influx of Ca^2+^ into the OK cells upon which a consequent translocation and activation of PKC*α* were absolutely dependent [40]. These findings are in agreement with the earlier studies of Tsimaratos et al. that showed PKC-induced activation of kidney Na^+^,K^+^-ATPase in C-peptide exposed tubular segments [21] (see above). Most importantly, these signalling events had a functional consequence with significant enhancement of proliferation seen in C-peptide treated cells [40]. All of these events were sensitive to PTX again providing evidence of the likely presence of a GPCR for C-peptide.

Most recently C-peptide has been found to promote translocation of the low molecular weight GTP-binding protein, Rho A, from the cytoplasm to the membrane of human kidney proximal tubular cells [41]. This effect was completely reliant on the upstream activation of phospholipase C (PLC). In fact, in these cells the activation of ERK, JNK, as well as PKC-*ε* and -*δ* was each sensitive to PLC inhibition, indicating an obligate dependency on upstream PLC activation by C-peptide. Once more, all stimulatory effects of C-peptide were PTX sensitive [41].

The molecular mechanisms by which C-peptide activates MAPK can now be described with some precision. The signal transduction pathway involves: (i) C-peptide binding to a PTX sensitive GPCR; (ii) the activation of PLC; (iii) subsequently increased diacylglycerol and intracellular Ca^2+^ levels stimulate several PKC isoforms; (iv) PKC-dependent activation and translocation of RhoA to plasma membrane; and (v) phosphorylation and activation of MAPKs.

## 6. C-PEPTIDE ACTIVATION OF PI-3-KINASE

The PI 3-kinases are a family of enzymes that phosphorylate the hydroxyl group at the 3 positions of the inositol ring of phosphatidyinositol. PI-3-kinases regulate a remarkably diverse range of cellular functions, including growth, proliferation, survival, differentiation, motility, and intracellular trafficking [42]. The PI-3-kinases are also a key component of insulin signalling pathways and this is of substantial interest in diabetes. Many of these functions of PI-3-kinase are related to their ablilty to activate protien kinase B (Akt) [42].

Robust C-peptide activation of PI-3-kinase has now been demonstrated by several authors in various cell types. As a result, it is known that physiological concentrations of C-peptide can regulate several aspects of PI-3-kinase signalling pathways in OK cells [40], Swiss 3T3 fibroblasts [37], SH-SY5Y neuroblastoma cells [43], human CD4^+^T cells [44] L6 myoblasts [45]. As a direct consequence, it is now recognised that stimulation of PI-3-kinase by C-peptide acting alone is responsible for (i) enhancement of neuronal and kidney tubular cell proliferation [40, 43]; (ii) increased T cell migration [44]; (iii) stimulation of peroxisome proliferators-activated receptor-*γ* (PPAR*γ*) in kidney tubule cells and associated gene transcription [46]; and (iv) upregulated glycogen synthesis in skeletal muscle cells [45].

## 7. EFFECTS OF C-PEPTIDE ON TRANSCRIPTION FACTORS

Given that many of the changes in cell phenotype that take place as a result of signalling by bioactive molecules are mediated by altered gene and protein expression, it is not surprising that C-peptide regulates activity of several key transcription factors.

Extending their observations of C-peptide activation of MAPKs Kitamura et al. described phosphorylation and activation of CREB, ATF-1, and ATF-2 in LEII cells [38]. Indeed 1 nM C-peptide and phorbol ester, a positive control, were equipotent in this regard. CREB and ATF proteins are transcription factors that bind when activated to specific response elements, CRE, in DNA thereby regulating transcription. Gel mobility shift assays clearly showed the binding of CREB to CRE in C-peptide treated cells. In the latter study, specific genes subject to regulation were not identified. However using neuroblastoma cells, it was reported that C-peptide treatment enhanced expression and translocation of nuclear factor-*κ*B (NF-*κ*B) and expression of the Bcl_2_ protein—an important mediator of the antiapoptotic effects governed by NF-*κ*B [43]. Control of NF-*κ*B by C-peptide in Swiss 3T3 has also been demonstrated. In these cells, 1 nM C-peptide activated transcription of cyclooxygenase-2 (COX-2) via NF-*κ*B consequent upon upstream activation of PKC [47]. COX-2, a cytokine inducible gene, is the rate-limiting enzyme in the conversion of arachidonic acid to prostaglandin, but the potential consequences of its upregulation by C-peptide remain unclear.

We performed detailed studies of transcription factor activation in OK proximal tubular cells [46, 48], comparing C-peptide effects to those of insulin and focussing on PPAR*γ* and NF-*κ*B. A member of the nuclear hormone receptor family, PPAR*γ* is the target for the insulin-sensitising thiazolidinediones currently used as therapeutic agents in the treatment of type 2 diabetes [49]. The expression of PPAR*γ* may also be regulated by insulin [50]. Using transient transfection of a peroxisome proliferator response element (PPRE)-luciferase reporter construct, we showed that both C-peptide and insulin transactivated PPRE via PPAR*γ* [46]. C-peptide (EC_50_ 4 nM) was more potent than insulin (EC_50_ 10 nM) in this regard, but both agents evoked phosphorylation of PPAR*γ* similarly via activation of PI-3-kinase. One consequence of PPAR*γ* activation by C-peptide and insulin was enhanced transcription of the prototypic PPAR*γ* regulated gene, CD36 [46]. Clearly, therefore, there is a degree of overlap between insulin and C-peptide signalling with both agent's effects being directed via PI-3-kinase towards PPAR*γ*. However, only the effects of C-peptide were attenuated by PTX and therefore C-peptide must be signalling through a receptor system fundamentally distinct from that of insulin. These data indicate an important novel mechanism whereby C-peptide and insulin may interact to regulate glycaemia and the expression of PPAR regulated genes such as those involved in metabolic control and inflammation.

The next step was to establish proof of principle that C-peptide had the capacity to act as a protective agent in diabetic nephropathy. To this end, we investigated whether C-peptide could counteract adverse effects precipitated by the administration of TNF-*α* to OK proximal tubular cells [48]. TNF-*α* is recognised as a major player in the development of diabetic nephropathy and may contribute to tubular cell apoptosis and tubular atrophy prominently observed in diabetic nephropathy [51–55]. TNF-*α* is a pleiotropic peptide cytokine, capable of eliciting a wide spectrum of cellular responses including differentiation, proliferation, inflammation, and cell death via interaction with two members of the TNF receptor family, TNF-R1 and TNF-R2 [54]. Predominantly produced by monocytes/macrophages but also by T and B-lymphocytes and glomerular mesangial cells [55, 56], TNF-*α* binding to TNF-R1 may simultaneously trigger apoptotic pathways by recruitment of death effector adaptor molecules with subsequent activation of caspase cascades, and antiapoptotic pathways by a pathway involving TNF receptor-associated factor2 (TRAF2) and nuclear factor-*κ*B (NF-*κ*B). Integration of these events determines the eventual cellular response to TNF-*α* stimulation. In particular NF-*κ*B stimulates transcription of antiapoptotic factors that modulate the caspase cascade, and thus NF-*κ*B activity acts as a checkpoint in a cell's decision to survive or apoptose in response to a given stimulus.

When applied to OK cells, TNF-*α* markedly reduced viability and induced apoptosis [48]. This was completely prevented by pretreatment with insulin or C-peptide. At the same time both insulin and C-peptide activated NF-*κ*B as judged by luciferase reporter assay. Insulin demonstrated a typical sigmoidal dose-response of NF-*κ*B activation, maximal at an applied concentration of 100 nM. On the other hand, C-peptide displayed completely different bell-shaped curve of NF-*κ*B stimulation, maximal with a 5 nM applied concentration. As in previous studies, PTX blocked only the C-peptide effect. However in these studies, the presence of a G*α*_*i*_-linked GPCR was revealed using an additional technique whereby C-peptide but not insulin was shown to stimulate GTP*γ*S binding to G*α*_*i*_ in OK cell membranes. In this work, the ability of C-peptide to prevent TNF-*α*-induced apoptosis appeared to be due to its ability to induce the expression, via NF-*κ*B activation, of survival genes such as TRAF2 [48].

This study provided evidence for the ability of C-peptide to regulate the expression of beneficial genes in the face of pathophysiological stimuli relevant to the development and pathology of diabetic nephropathy. Again, C-peptide appeared to be acting via G*α*_*i*_, and although C-peptide shared some signalling properties with insulin it clearly possessed its own unique signalling capabilities.

## 8. IS C-PEPTIDE AN INSULIN MIMETIC?

There is no doubt that some components of C-peptide signalling pathways are shared with those of insulin. For example, activations of MAPKs and PI-3-kinase are well described downstream events following insulin-insulin receptor interactions [57]. In the absence of a fully characterised C-peptide receptor, and given that C-peptide exhibited some insulin-like actions such as increasing muscle glucose transport [58, 59], Grunberger et al. wondered whether C-peptide signalling may simply be explained by activation of the insulin receptor signalling system. They found in L6 myoblasts that C-peptide (0.3–3 nM) activated insulin receptor tyrosine kinase, tyrosine phosphorylation of the insulin receptor substrate-1 (IRS-1), MAPK, PI-3-kinase, p90 ribosomal S6 kinase (p90RSK) and glycogen synthase kinase-3 (GSK3), and glycogen synthesis [45]. Submaximal concentrations of insulin and C-peptide were additive in effect, whereas maximal concentrations were not. C-peptide stimulation of glycogen synthesis was not abrogated by PTX, although earlier specific steps in the signalling pathway were not tested in this way [45]. These findings contrasted with those of Zierath et al. who found no evidence of insulin receptor activation by C-peptide in human skeletal muscle [59]. Nonetheless their results led Grunberger et al. to speculate that, amongst other possibilities, C-peptide per se may activate the insulin receptor [45].

There are several lines of evidence against this however. The most compelling is the description by multiple authors of the specific PTX sensitivity of C-peptide effects, and most recently the observation that C-peptide provokes GTP*γ*S binding to G*α*_*i*_, [48] an observation compatible with guanine nucleotide exchange occurring on a G-protein after ligation of a GPCR. Furthermore, the dose-response curves for insulin are typically sigmoidal whereas those for C-peptide are often bell shaped with a diminution of activity at higher concentrations. At least in vitro C-peptide appears more potent on a molar basis than insulin. Typically C-peptide signalling effects are apparent at midhigh pM concentrations, maximal at *∼*1–5 nM and thereafter decline. In contrast, insulin responses generally become evident at *∼*10 nM increasing up to 100–1000 nM where they plateau. Finally, using plasmon resonance no interaction of C-peptide with soluble purified insulin receptor A or insulin-like growth factor receptor was detected in a very recent study [60].

The balance of evidence suggests that C-peptide mediates its own distinct signalling through a GPCR. There is certainly some overlap between the signalling pathways activated by C-peptide and insulin but this is a common observation in biological systems. Crosstalk between GPCR activated signalling and receptor tyrosine kinase activated pathways occurs by transactivation. This well-described mechanism of cross-communication between signalling systems enables the cell to integrate multiple signals derived from its environment. Transactivation of the epidermal growth factor receptor following agonist stimulation of G*α*_*i*_ or G*α*_*q*_ coupled GPCRs represents the paradigm for this system, however similar events have now been described for the platelet derived growth factor receptor, the insulin-like growth factor-1 receptor, and IRS-1.

## 9. SUMMARY AND CONCLUSION

In the last 10 years, there has been a revolution in thinking about C-peptide. There can now be no doubt that C-peptide truly exerts a variety of biological effects. These effects are underpinned by solid evidence of cell signalling events (summarised in Table 1), most likely mediated via a specific GPCR. The concentrations of C-peptide required to elicit signalling outputs are entirely congruent with what is known about binding affinities of C-peptide for its putative receptor. It should be acknowledged however that the lack of detailed identification/cloning of a receptor remains a weakness in the field. Nonetheless, the importance of these signalling effects cannot be underestimated. The pathways stimulated are fundamental to cell function and phenotype, impacting on such basic processes as life and death. The next challenge is to identify the receptor and to bring C-peptide nearer to the clinical arena in order to test its potential benefits in ameliorating the devastating consequences and complications of diabetes.

## Figures and Tables

**Table 1:** Signalling elements activated by C-peptide according to cell type studied (for abbreviations see text).

Cell type studied								
Signalling element	Kidney tubular	Muscle	Fibroblasts	Endothelial	Erythrocytes	Neuroblastoma	CD4 + T Cells	Nerve
Na^+^,K^+^-ATPase	+				+			+
↑[Ca^2+^]_*i*_	+			+				
PKC	+		+					
NO release		+		+				
ERK	+	+	+	+				
p38 MAPK				+				
JNK	+							
PLC	+							
p90RSK		+						
GSK3		+						
PI3K	+	+		+		+	+	
CREB				+				
NF-*κ*B	+		+			+		
PPAR*γ*	+							
